# The effect of total glucoside of paeony on gut microbiota in NOD mice with Sjögren’s syndrome based on high-throughput sequencing of 16SrRNA gene

**DOI:** 10.1186/s13020-020-00342-w

**Published:** 2020-06-11

**Authors:** Wen-Wen Lu, Tian-Xiao Fu, Qing Wang, Yi-Lian Chen, Tian-Yi Li, Guo-Lin Wu

**Affiliations:** 1 Department of Traditional Chinese Medicine, The First Affiliated Hospital, College of Medicine, Zhejiang University, Hangzhou, 310003 China; 2 Internal Medicine, Tongde Hospital of Zhejiang Provincial, Hangzhou, 310012 China; 3 Basic Medical College, Zhejiang University of Chinese Medicine, Hangzhou, 310053 China

**Keywords:** Total glucoside of paeony, Sjögren’s syndrome, Microbiota, NOD mice, 16SrRNA

## Abstract

**Purpose:**

To investigate the effect of total glucoside of paeony (TGP) on gut microbiota in NOD mice with Sjögren’s syndrome (SS), using high-throughput sequencing of 16SrRNA gene.

**Methods:**

Twenty-four NOD mice were randomly assigned to 4 groups (n = 6 per group): sham group receiving deionized water (0.4 ml), hydroxychloroquin group receiving hydroxychloroquin (0.4 ml), TGP group receiving TGP (0.4 ml), and TGP + hydroxychloroquin group receiving 0.4 ml TGP and 0.4 ml hydroxychloroquin. Balb/c mice (n = 6) receiving 0.4 ml deionized water were used as a control group. After intragastric injection of drugs for 8 weeks, feces were collected for high-throughput sequencing of 16SrRNA gene.

**Results:**

The sequencing of 16SrRNA gene resulted in 3686 OTUs, and 10 phyla and 69 genera were identified. Compared with the control group, the indices of Chao, Ace and Shannon in the other 4 groups were significantly lower (P < 0.05), and the Simpson index were significantly higher in the TGP, hydroxychloroquine, and sham groups (P < 0.05). Compared with the sham group, the indices of Chao, Ace and Shannon were significantly higher (P < 0.05), whereas the Simpson index was significantly lower (P < 0.05) in the TGP and TGP + hydroxychloroquine groups. At phylum level, Bacteroidetes was least abundant (36.1%), and Firmicutes was most abundant (56.28%) in the TGP + hydroxychloroquine group. Compared with the other 4 groups, Bacteroidetes was significantly less abundant (P < 0.05) and Firmicutes was significantly more abundant (P < 0.05) in the TGP + hydroxychloroquine group. Verrucomicrobia was most abundant (12.26%) in the hydroxychloroquine, and was significantly more abundant compared with the other 3 groups (P < 0.05). At genus level, compared with the control group, the abundance of Lactobacillus and Incertae of Phylum Firmicutes and Desulfovibrio of Phylum Proteobacteria was significantly increased, and the abundance of Bacteroides and Alloprevotella of Phylum Bacteroidetes and Pseudoflavonifractor of Phylum Firmicutes was significantly decreased in the TGP + hydroxychloroquine group (P < 0.05). Compared with the hydroxychloroquine group, the abundance of Akkermansia of Phylum Verrucomicrobia was significantly decreased in the TGP and TGP + hydroxychloroquine groups (P < 0.05). The abundance of Alistipes of Phylum Bacteroidetes and Desulfovibrio of Phylum Proteobacteria was significantly increased in the TGP + hydroxychloroquine group (P < 0.05).

**Conclusions:**

TGP increases the growth of many key beneficial bacteria, inhibits the growth of dominant pathogenic bacteria, and increases the diversity and abundance of gut microorganisms, especially when combined with hydroxychloroquine. Our findings suggest that TGP may be effective to treat SS by improving the microecological structure of the gut.

## Background

Sjögren’s syndrome (SS) is a group of systemic autoimmune diseases with high heterogeneity. In clinic, salivary gland and lacrimal gland are often involved. In severe cases, visceral organs may be involved and multiple system injuries may occur. To date, the etiology and pathogenesis of SS have not been fully understood. Recently, more and more attentions have been paid to study the association between intestinal microorganisms and diseases. Approximately 1500 bacterial floras in the intestinal tract have been found to be associated with the pathogenesis of many diseases such as metabolic diseases, digestive tract diseases, and autoimmune-related diseases. Intervention of gut microbiota can treat and even prevent many diseases [1]. Several studies have found that the pathogenesis of SS is associated with gut microecological disorder [2, 3].

Total glucoside of paeony (TGP) is the main active ingredient that is extracted from traditional Chinese medicine Paeonia Alba. There are several components in TGP, such as paeoniflorin, hydroxy paeoniflorin, paeoniflorin, albiflorin,benzoyl paeoniflorin, and so on. Among them, paeoniflorin accounts for more than 90% of the total glycosides [4]. TGP is widely used in the treatment of rheumatic immune diseases, and is associated with good clinical outcomes in patients with SS. TGP might be beneficial for patients with RA and in reduction of adverse effects, compared with no treatment [5]. TGP appears to improve the glandular secreting function and decrease the level of inflammatory cytokines [6].

The pathological changes of submandibular gland of NOD mice are similar to those of SS, and thus NOD mice have been commonly used as an experimental animal model of SS [7]. NOD mice found inflammation of the saliva and lacrimal glands, the incidence of female mice was significantly higher, and the earliest evidence of autoimmunity occurred at 6 to 7 weeks of age [7].

Hydroxychloroquine is a highly safe immunosuppressant, it can significantly improve the symptoms of dry mouth, dry eyes, arthralgia and other symptoms in patients with Sjogren’s syndrome, and can reduce the erythrocyte sedimentation rate (ESR) and C-reactive protein (CRP), Immunoglobulin (Ig)G, IgM [8]. It is recommended in the British Rheumatology Society [9] and Chinese Rheumatology Society guidelines on the diagnosis and treatment of Sjogren’s syndrome [10]. Therefore, in this study, hydroxychloroquine was used as a positive control group.

We have previously found that TGP regulated the balance between Th1 and Th2 immune responses in NOD mice, increased the mRNA and protein expression of AQP-5 in the submandibular gland, and reduced the pathological damage of the submandibular gland of NOD mice [11, 12]. In this study, we performed high-throughput sequencing of the 16SrRNA gene to investigate the changes of gut microbiota in NOD mice and studied the regulatory effect of TGP. The purpose of this study was to explore the mechanism of TGP in the treatment of SS from the perspective of intestinal microecology.

## Materials and methods

### Animals

Twenty-four NOD female mice (age, 8-week old; body weight, 200 ± 20 g) [7] and 6 control Balb/c mice were used in this study. Animals were housed at room temperature (25 °C) with 46% humidity and a 12 h light/dark cycle in the Animal Center of Zhejiang Chinese Medical University. The animals were fed standard animal chow and water in the specific pathogen-free (SPF) room. All animals were purchased from Shanghai Sippe-Bk Lab Animal Co., Ltd, Shanghai, China (No. 20130016000707 and 20130016000706).

### Experimental drugs

TGP capsule was produced by Ningbo Lihua Pharmaceutical Co., Ltd. (Batch No: H20055058; 0.3 g/tablet). The TGP capsule was diluted with distilled water to a mixture with 13.5 mg/ml crude drug. Hydroxychloroquine sulfate tablets were produced by Shanghai Shangyao Zhongxi Pharmaceutical Co., Ltd, Shanghai, China (Batch No: h199902630; 1 g/tablet), and were diluted with distilled water to a mixture with 3 mg/ml crude drug.

### Animal groups and drug administration

After 1 week adaptation, 24 NOD mice were randomly assigned to 4 groups (n = 6 mice per group): the sham group receiving deionized water (0.4 ml), hydroxychloroquin group receiving hydroxychloroquin (0.4 ml), TGP group receiving TGP (0.4 ml), and TGP + hydroxychloroquin group (Combination group) receiving 0.4 ml TGP and 0.4 ml hydroxychloroquin. Additional 6 age-matched Balb/c mice receiving 0.4 ml deionized water were used as a control group. TGP, hydroxychloroquin, or deionized water (0.4 ml) was intragastrically injected once daily for 8 weeks. During the experimental period, mice were allowed to freely access to food and water. The animals were fasted with food but not water after the last intragastric injection, and were sacrificed next day. After the colon was removed, the intestinal tissues and fresh feces on the intestinal tract were collected immediately, and stored in a 1.5 ml centrifuge tube of high pressure sterilization at − 80 °C.

### 16SrRNA gene sequencing of gut microbiota

#### DNA extraction and PCR amplification

Genomic DNA was extracted from mouse feces using the DNA extraction kit according to the manufacturer’s instructions. The barcoded primers targeting the designated sequencing region were synthesized to amplify the v3-v4 region of the bacterial 16SrRNA gene. The primers used were as follows: 341F (5′-CCTACGGGNGGCWGCAG-3′) and 785R (5′-GACTACHVGGGTATCTAATCC-3′). PCR was performed in a reaction mixture containing 12.5 μL 2 × KAPA HiF HotStart ReadyMix (KAPA Biosystems), 0.25 μL primers (25 mmol/L), 1 μL DNA, and 9.5 μL ddH_2_O. The reaction condition was as follows: initial denaturation at 95 °C for 2 min; denaturation at 95 °C for 30 s, annealing at 55 °C for 30 s, and extension at 72 °C for 30 s with 25 cycles; and final extension at 72 °C for 5 min. Each sample was repeated twice.

#### DNA purification and sequencing

DNA amplicons were analyzed with 2% agarose gel electrophoresis, and the target fragments were purified by QIAquick Gel Extraction Kit (Qiagen). Qubit Fluorometer (Thermo Fisher Scientifc) was used to detect the DNA mass concentration and DNAs with a concentration of higher than 1.0 ng/L was selected. QsEquation 100 DNA analyzer (Bioptic) was used to detect the length distribution of bacterial DNA library, and DNA fragments with the length of the target fragment, single peak, and no adaptor sequence were selected. DNA molar concentrations were detected using Kapa library Quantification Kit, and used as the standard for mixing DNA libraries. Qualified DNAs were mixed and denatured, and used for high-throughput parallel sequencing on an Illumina Miseq sequencing instrument (Illumina). During the sequencing, the two ends (namely paired end) of the library were sequenced.

#### Sequence processing

Flash software was used to merge raw sequences to obtain merged sequences and the length distribution. The merged sequences were filtered using Mothur. The filtering criteria were as follows: the sequences with average quality score ≤ 20; the sequences with N; the sequences with too long homopolymer (> 10 bp); the sequences with many primer mismatches (≥ 4 bp); the primer sequences; too short (< 200 bp) and too long (≥ 500 bp) sequences; and the chimera in comparison with gold data set by Uchime software.

#### OTU taxonomic classification

Based on the sequence similarity of 97%, the sequences were clustered into OTU (operational taxonomic unit). After the rare sequences were removed, OTU-taxa table was generated, and ACE, Chao, Shannon and Simpson indices were calculated. All sequences were blasted against the SILVA database, using a RDP classifier. Based on Bergey’s taxonomy, the OTU sequences are classified into six levels: kingdom, phylum, class, order, family and genus.

### Statistical analysis

Metastats was used to analyze the differences in the relative abundance of phylum and genus, and the p value and q value between groups was calculated. All data were expressed as mean ± standard deviation (± s). Statistical analysis was performed using SPSS19.0 statistical software. Data with normal distribution and homogeneous variance were analyzed using one-way ANOVA. Data without normal distribution were analyzed using rank sum test. P < 0.05 was considered statistically significant.

## Results

### Summary of sequencing results

**Fig. 1:**
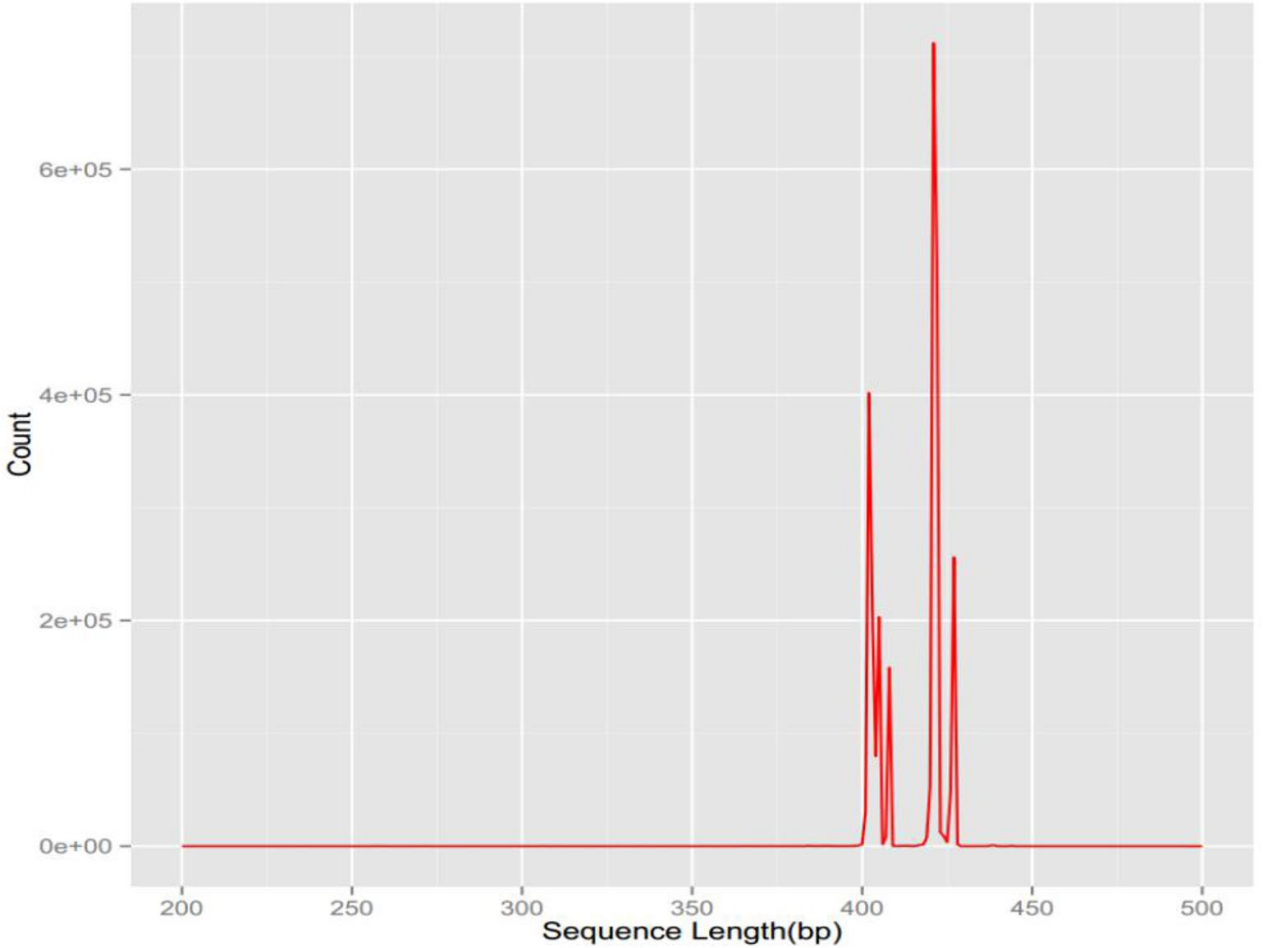
The sequence length distribution after filtering in each group

The final high-quality sequence was obtained by high-throughput sequencing of intestinal stool samples of NOD mice using Illumina Miseq sequencing platform which ranged from 233,434 to 378,201. The ratio of high-quality sequence to effective sequence was > 95%, and the average length ranged 401–428 bp. According to the sequence similarity, the sequences were classified into OTUs. The number of OTUs ranged from 608 to 1107 in each treatment group. The biological abundance was high and the variance was great (Table 1, Fig. 1).

**Table 1:** The number of OTUs and sequences (n = 6)

Groups	Effective sequence/strip	High-quality sequence/strip	Quantity of OTU
Combination group	259452	253145	608
TGP group	262606	256472	629
Hydroxychloroquin group	326389	318783	630
Sham group	265057	259065	712
Control group	245261	233434	1107

### The distribution of gut microbial abundance

**Fig. 2:**
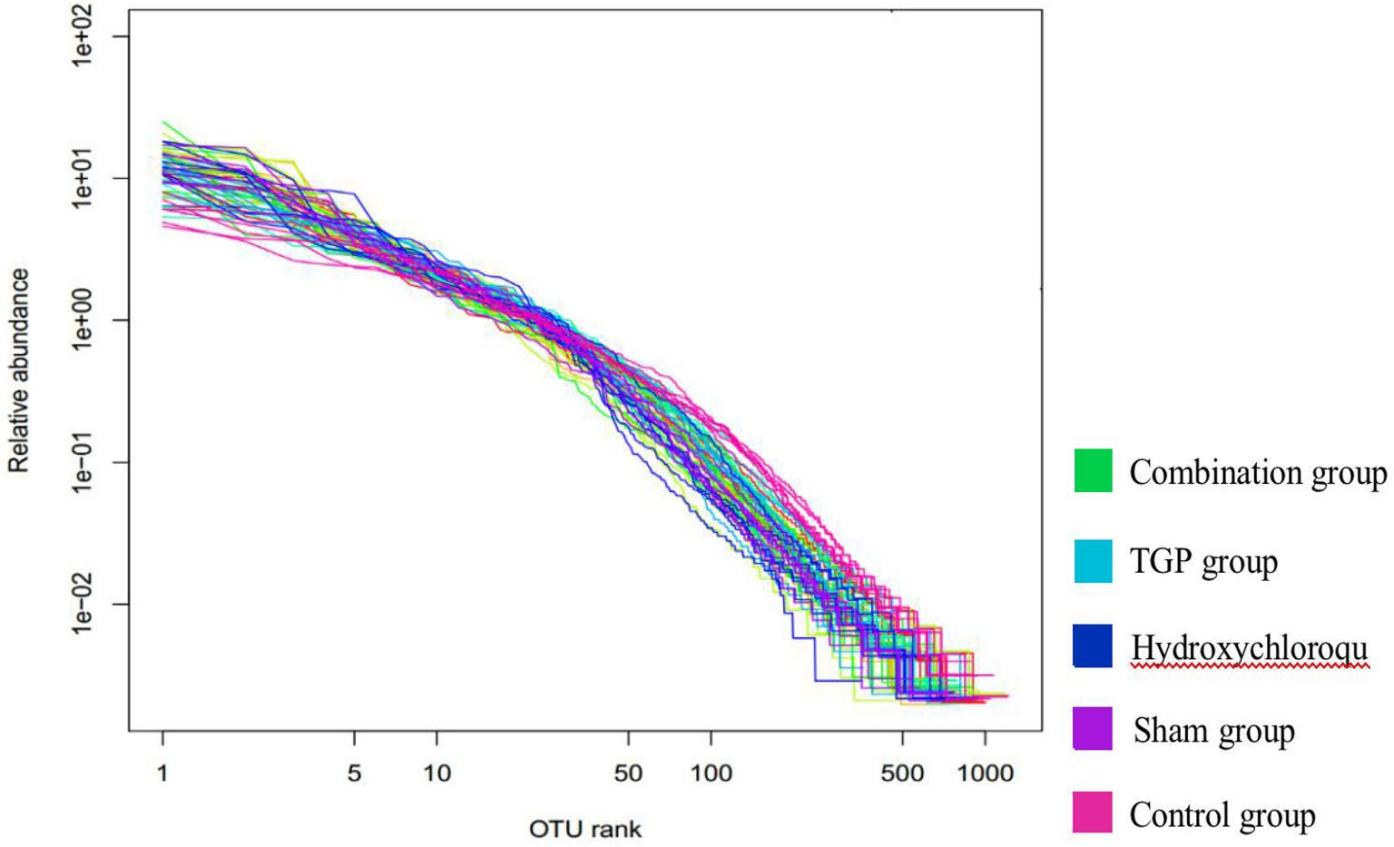
The gut microbial abundance curve of each group

Figure 2 shows the abundance distribution curve of each group using the logarithm (log2) of the abundance of OTUs. The results showed that the sample in each group had sufficient abundance, since the number of abundance ranged from 500 to 1000 for most samples. In addition, the curve exhibited flat end, suggesting that the distribution of gut bacteria was uniform and the samples was suitable for diversity analysis.

### Analysis of alpha diversity of gut microbiota

Table 2 summarizes the analysis of alpha diversity of gut microbiota in each group. The richness and diversity of gut microbiota were assessed using alpha diversity. Chao and Ace indices were used to reflect the abundance of gut microbiota in the sample, and Shannon and Simpson indices were used to reflect the diversity of gut microbiota. Coverage index was used to reflect the coverage rate of each sample library. A sequence with a high coverage index indicated that this sequence had a high probability to be detected. The coverage index can directly reflect whether the sequencing results represent the true situation of gut microbiota. In this study, all samples had a Coverage index of > 0.99, which met the requirement for bacterial diversity analysis.

**Table 2:** The analysis of Alpha diversity index of gut microbiota (± s, n = 6)

Groups	Chao	Ace	Shannon	Simpson
Combination group	1040.10 ± 124.89^a^	1072.86 ± 140.70^a^	4.47 ± 0.27^a^	0.03 ± 0.01
TGP group	1071.76 ± 224.84^a^	1100.85 ± 206.99^a^	4.29 ± 0.23^a^	0.03 ± 0.00^a^
Hydroxychlo-roquin group	997.11 ± 247.89^a^	1016.46 ± 206.29^a^	3.99 ± 0.34^a,b^	0.05 ± 0.02^a,b^
Sham group	929.60 ± 122.15^a^	1025.19 ± 73.90^a^	3.99 ± 0.19^a,b^	0.05 ± 0.01^a,b^
Control group	1357.10 ± 148.57	1450.94 ± 153.07	4.93 ± 0.15	0.02 ± 0.00

^a^P < 0.05 vs control group

^b^P < 0.05 vs TGP and TGP + hydroxychloroquine group

Compared with the control group, the indexes of Chao, Ace, and Shannon of the sham operation group and the other three groups were all significantly reduced (p < 0.05), and the Shannon index was significantly increased (P < 0.05). Compared with the sham group and the hydroxychloroquine group, the Shannon index of the TGP and TGP + hydroxychloroquine groups was significantly increased (P < 0.05). There was no significant difference in Shannon index between the TGP group and the TGP + hydroxychloroquine group (P > 0.05).

**Fig. 3:**
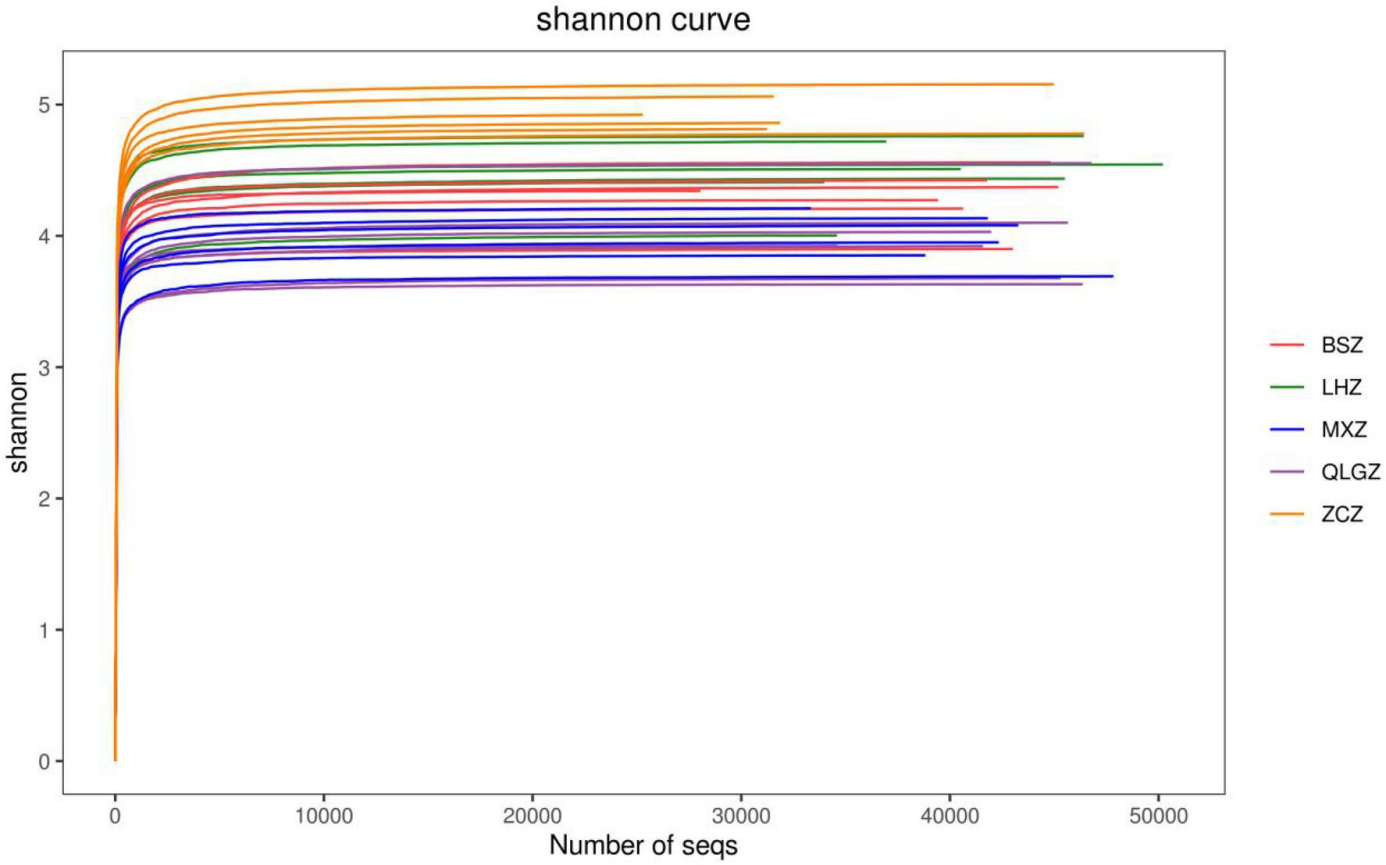
The gut intestinal species dilution curve and Shannon curve of each group. LHZ, combination group; BSZ, TGP group; QLGZ, hydroxychloroquin group; MXZ, Sham group; ZCZ, control group

**Fig. 4:**
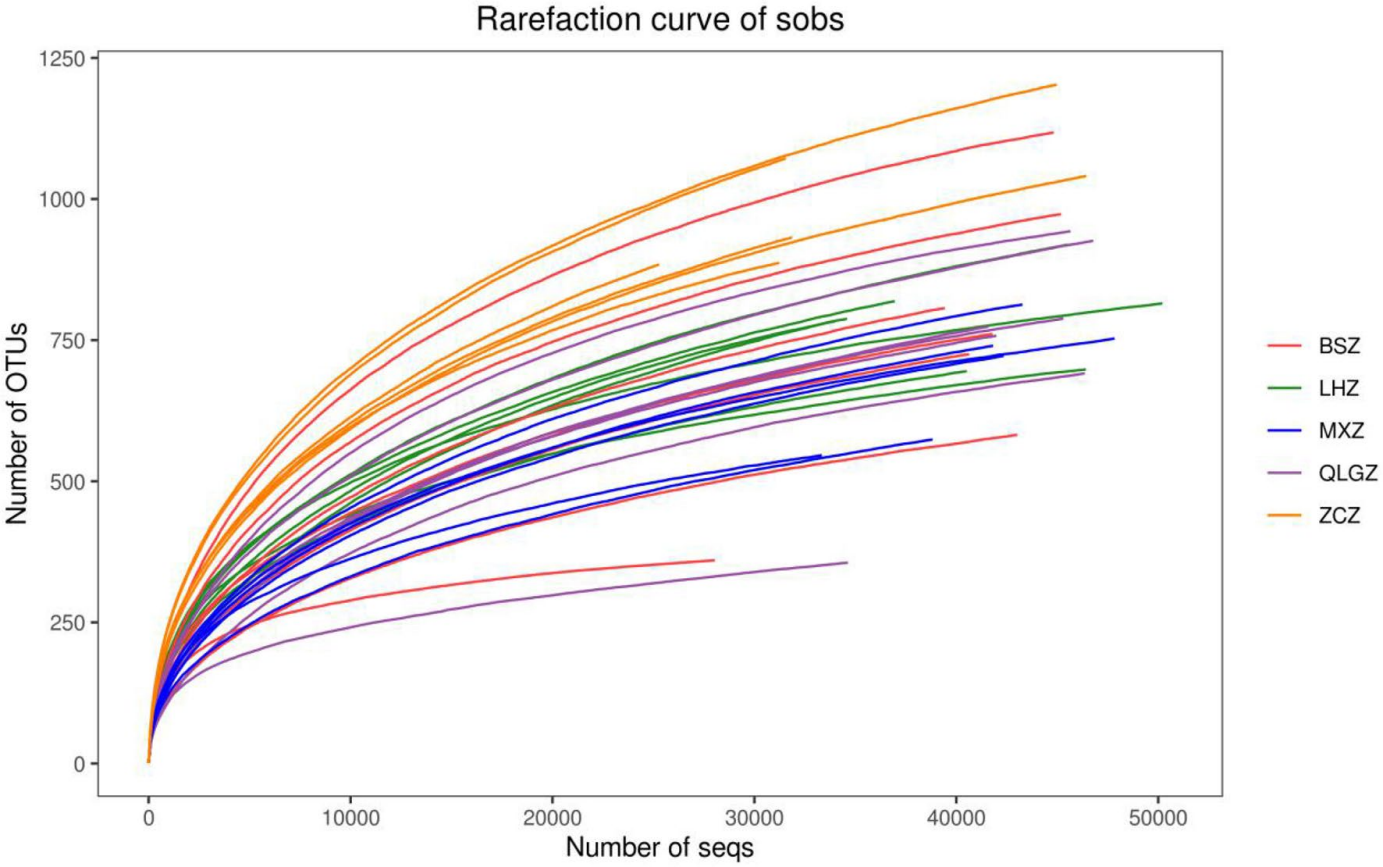
The gut intestinal species dilution curve and Shannon curve of each group. LHZ, combination group; BSZ, TGP group; QLGZ, hydroxychloroquin group; MXZ, Sham group; ZCZ, control group

The Simpson index of the sham group and hydroxychloroquine group was significantly higher than that of the TGP and TGP + hydroxychloroquine groups (p < 0.05). There was no significant difference between the TGP group and the TGP + hydroxychloroquine group (P > 0.05) (Figs. 3 and 4).

### The abundance analysis of the structure of gut microbiota

#### The abundance analysis of the structure of gut microbiota at phylum level

**Fig. 5:**
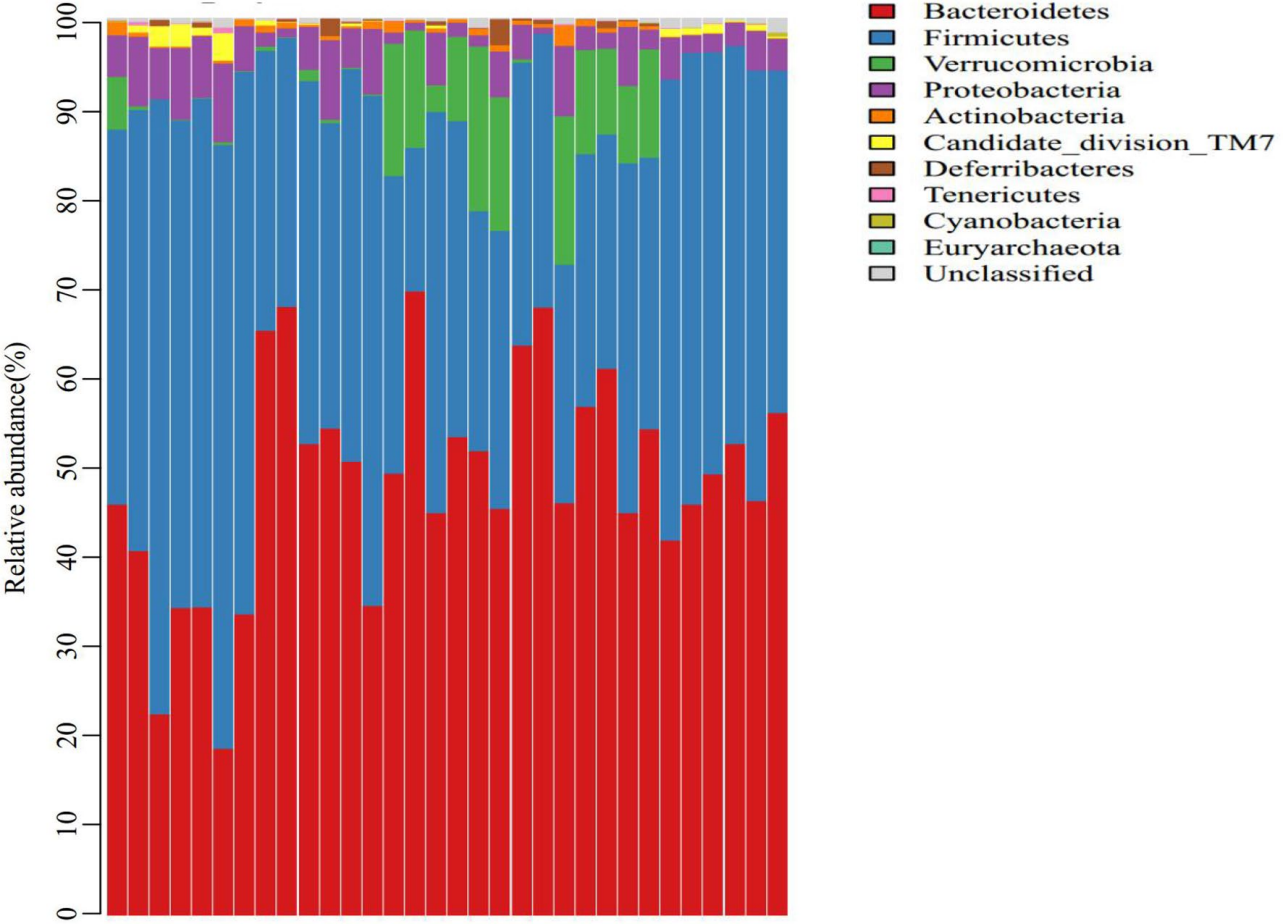
The abundance analysis of the structure of gut microbiota at phylum level in each group. LHZ, combination group; BSZ, TGP group; QLGZ, hydroxychloroquin group; MXZ, Sham group; ZCZ, control group

**Fig. 6:**
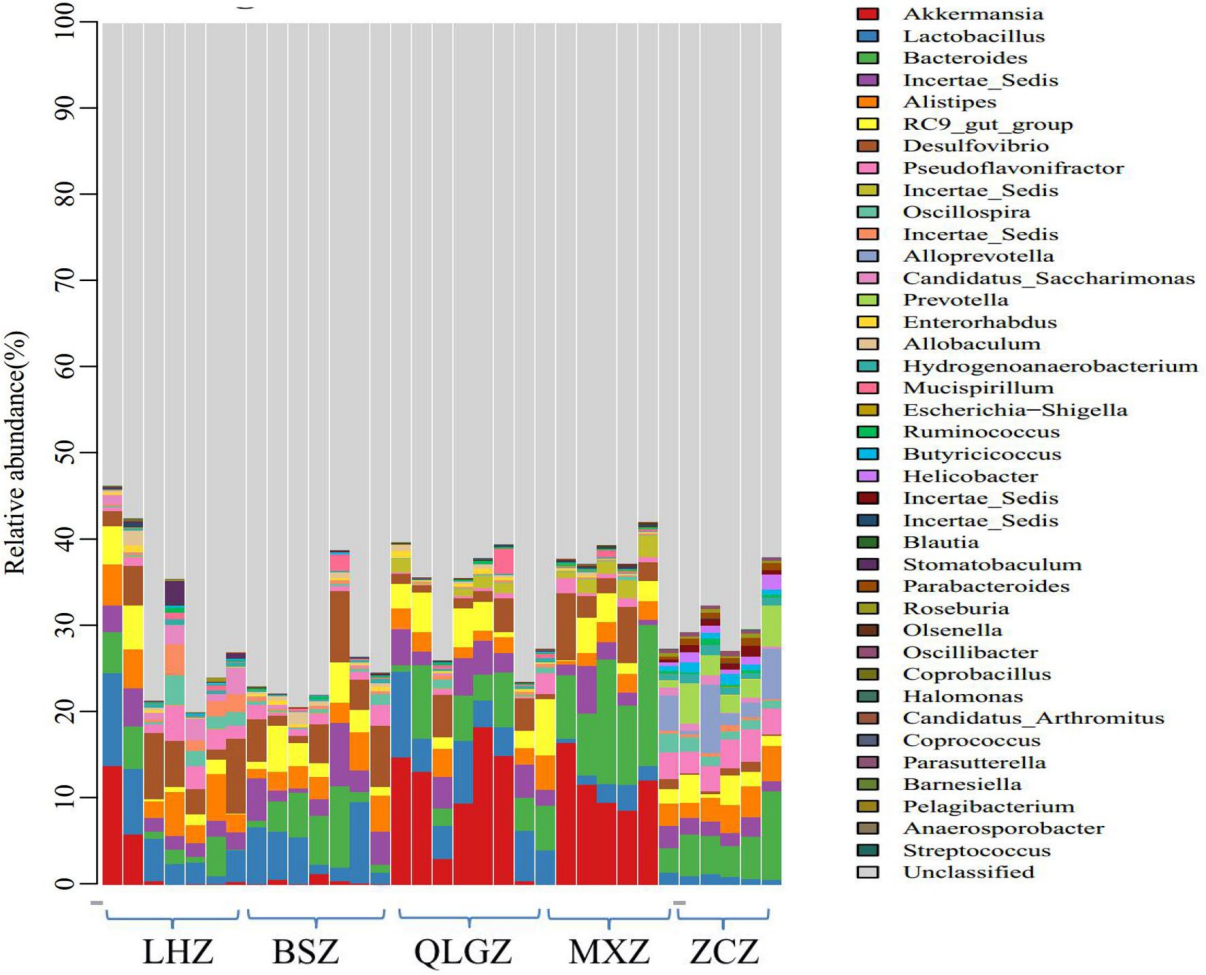
The abundance analysis of the structure of gut microbiota at genus level in each group. LHZ, Combination group; BSZ, TGP group; QLGZ, hydroxychloroquin group; MXZ, Sham group;ZCZ, control group

**Fig. 7:**
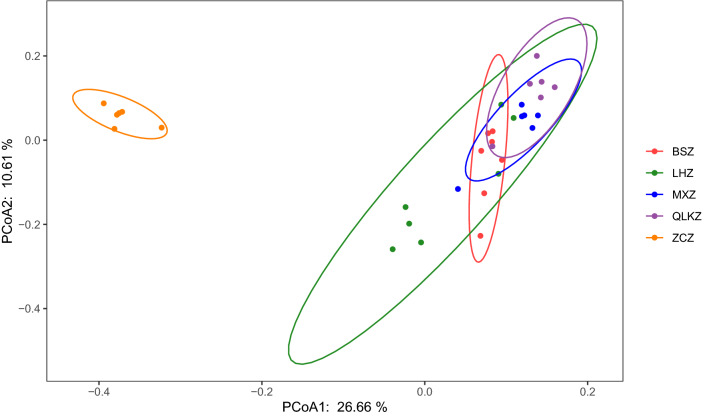
PCoA analysis in each group. LHZ, combination group; BSZ, TGP group; QLGZ, hydroxychloroquin group; MXZ, Sham group; ZCZ, control group

To compare the effect of TGP, hydroxychloroquine or their combination on the structure and composition of gut microbial community, we performed a comparative study on the clustering of OTUs at the levels of phylum and genus. Ten phyla and 69 genera were identified in the samples. The 10 phyla included Euryarchaeota, Actinobacteria, Bacteroidetes, Candidate division TM7, Cyanobacteria, Deferribacteres, Firmicutes, Proteobacteria, Tenericutes, and Verrucomicrobia. Bacteroidetes and Firmicutes were most abundant in the microbial community, accounting for 88.46% of all samples (47.29% for Bacteroides and 41.17% for Firmicutes). Compared with control group, Bacteroidetes was more abundant (55.06%) and Firmicutes was less abundant (30.07%) in the sham group (P > 0.05), Verrucomicrobia was more abundant (9.75%) in sham groups (P < 0.05). In the TGP + hydroxychloroquine group, Bacteroidetes was least abundant (36.1%), and Firmicutes was most abundant (56.28%). Compared with the other 4 groups, Bacteroidetes was significantly less abundant in the TGP + hydroxychloroquine group (P < 0.05), and Firmicutes was significantly more abundant in the TGP + hydroxychloroquine group (P < 0.05). However, there were no significant differences in the abundance of Bacteroidetes and Firmicutes between the hydroxychloroquine and sham groups (P > 0.05), and between the TGP and sham groups (P > 0.05). Verrucomicrobia was most abundant in the hydroxychloroquine (12.26%), and was significantly more abundant compared with the other 3 groups (P < 0.05). There were no significant differences in the abundance of other phyla among these groups (Figs. 5, 6, 7).

#### The abundance analysis of the structure of gut microbiota at genus level

We then compared the abundance of the structure of gut microbiota at genus level. Compared with control group, the abundance of Akkermansia of Phylum Verrucomicrobia was significantly increased in the sham group and hydroxychloroquine group (P < 0.05). Compared with the control group, the abundance of Lactobacillus and Incertae of Phylum Firmicutes and Desulfovibrio of Phylum Proteobacteria was significantly increased, and the abundance of Bacteroides and Alloprevotella of Phylum Bacteroidetes and Pseudoflavonifractor of Phylum Firmicutes was significantly decreased in the TGP + hydroxychloroquine group (P < 0.05). Compared with the sham group, the abundance of Lactobacillus of Phylum Firmicutes was significantly increased, and the abundance of Bacteroides of Phylum Bacteroidetes and Akkermansia of Phylum Verrucomicrobia was significantly decreased in the TGP and TGP + hydroxychloroquine groups (P < 0.05). In addition, the abundance of Alistipes of Phylum Bacteroidetes was significantly increased in the TGP + hydroxychloroquine group (P < 0.05).

Compared with the hydroxychloroquine group, the abundance of Akkermansia of Phylum Verrucomicrobia was significantly decreased in the TGP and TGP + hydroxychloroquine groups (P < 0.05). The abundance of Alistipes of Phylum Bacteroidetes and Desulfovibrio of Phylum Proteobacteria was significantly increased in the TGP + hydroxychloroquine group (P < 0.05).

### The analysis of the composition of gut microbiota

PCA (principal coordinates analysis) was used to analyze the composition of gut microbiota. PCA analyzed OTU data of different samples using ANOVA, and reflected the distance and difference between samples on the two-dimensional coordinate map. In PCoA plots, different samples exhibited the clustering or scattering distribution, and the samples with similar composition were closer to each other in the plot.

The control group was far away from the sham group, indicating that the intestinal flora of the sham group had changed compared with the control group. The distance between the sham group (purple circle) and the hydroxychloroquine group (blue circle) was very close and couldn’t be distinguished in the PCoA diagram, indicating that hydroxychloroquine has little effect on the composition of NOD mice intestinal flora. Compared with the control group, the TGP group and TGP + hydroxychloroquine group exhibited similarity to the control group at PC2, but showed dissimilarity to the control group at PC1. Therefore, PC1 was used to distinguish the difference between the TGP group, TGP + hydroxychloroquine group and the control group.

## Discussion

In China, the incidence rate of SS in China is 0.33% to 0.77%, and the incidence rate of females is high, especially in women of childbearing age, the ratio of female to male is 21: 1; the average age of onset is 44.6 ± 12 [13]. To date, the pathogenesis of SS is still unclear, and there is no specific therapy for the treatment of SS in the clinical practice. Western medicine adopts local replacement therapy to relieve symptoms of SS, and hormones or immunosuppressants are added to delay the autoimmune response when multiple tissues and organs are involved [14]. However, many adverse reactions are associated with the long-term use of Western medicine, especially hormones, resulting in a poor quality of life of patients with SS. As one of the main drugs for rheumatic diseases,. The effective monomers of Paeony are mainly a group of glycosides, which are collectively referred as total glucosides of paeony. Paeoniflorin accounts for 90% of the total glucosides. Modern pharmacological studies have found that TGP regulates immune responses via Th, Treg, and many cytokines, and produces anti-inflammatory, anti-oxidative, analgesic and liver-protecting effects. In addition, TGP is effective on the treatment of rheumatoid arthritis, and has a promising application in the treatment of autoimmune diseases [15].

In recent years, several studies had found that intestinal microecology could affect the local intestinal mucosal barrier by activating a large number of inflammatory factors, inhibiting the immune function of normal mucosa, and producing autoantibodies, thus leading to a variety of immune diseases such as systemic lupus erythematosus and ankylosing spondylitis [16]. The diversity of gut bacterial flora was the basis of the absorption of drugs and nutrients, maintenance of metabolism and regulation of the immune barrier. The changes in the diversity of gut bacterial flora will inhibit the innate immunity of the host, thus affecting the response of the body to the disease [17]. In this study, we performed high-throughput sequencing of the 16SrRNA gene to investigate the diversity and structure of gut microbiota in NOD mice, and to examine the regulatory effect of TGP. Our results showed that the number of OTUs in the control group was significantly higher than that in the other groups, suggesting that the abundance of gut microbiota was the highest in the natural state, and the variation within the group was small. In the alpha diversity analysis, the coverage index is close to 1, suggesting that there was a high bacterial coverage rate in the sequencing samples with a moderate depth and comprehensive bacterial species. Chao, ACE and Shannon indices of the control group were the highest among groups, suggesting that the abundance of gut microbiota was the best and the diversity was the largest in the non-intervention samples. The values of Chao and ACE indices in the drug intervention groups were similar, suggesting that gut bacteria in each sample was relatively rich. Although the abundance of gut microbiota was higher in the TGP and hydroxychloroquine groups than in the sham group, there was no significant difference among these groups, suggesting that TGP and hydroxychloroquine may slightly improve the abundance. No statistical significance was found among these groups may be due to the inability of the abundance of gut microbiota to return to the normal level after treatment. In addition, these results also suggested that the abundance of gut microbiota was greatly affected in NOD mice under the state of SS. The Shannon index in the TGP and TGP + hydroxychloroquine groups was significantly greater than in the hydroxychloroquine group, suggesting that the addition of TGP improved the diversity of gut microbiota. In addition, the Simpson index, which represents the low diversity of gut microbiota, was significantly lower in NOD mice, suggesting that the diversity of gut microbiota in SS mice was reduced. Although hydroxychloroquine slightly improved the diversity of gut microbiota, no statistical significance was found. The addition of TGP significantly changed the structure of gut microbiota, and improved the abundance of gut microbiota to the level similar to the control group. Dilution curve and Shannon curve also demonstrated that the new strains were not found and the diversity of gut microbiota was not changed with increased sequencing. Since we have previously found that TGP can reduce inflammatory cytokines and regulate Th1/Th2 immune balance, we speculate that the effect of TGP may be caused by increasing the abundance and diversity of gut microbiota, correcting dysfunctional structure of gut microbiota and improving the immunity.

In the analysis of the structure of gut microbiota at phylum level, we found that Bacteroidetes (47.29%) and Firmicutes (41.17%) were the most abundant in the microbial community. This finding was consistent with the previous report by Bezirtzoglou et al. [18], showing that Bacteroidetes and Firmicutes are the dominant bacterial flora in human intestine. In addition, the ratio of Firmicutes to Bacteroidetes (F/B ration) is commonly used to evaluate the imbalance of intestinal microflora, and the F/B ration is significantly correlated with the composition of the intestinal microflora [19, 20]. In many diseases such as obesity, hypertension, inflammatory bowel disease, rheumatoid arthritis, and systemic lupus erythematosus, the F/B ratio is lower than the normal level. Consistent with these reports, our findings showed that Bacteroides were most abundant in the sham group, followed by the hydroxychloroquine group, whereas Firmicutes was most abundant in the TGP + hydroxychloroquine group, followed by the control group. The abundance analysis at phylum level showed that Firmicutes was inhibited in the gut of SS mice, and Bacteroides became conditional pathogens, which promoted inflammatory reactions. TGP treatment recovered the structure of gut microbiota by increasing the abundance of Firmicutes, thus resulting in inhibition of the pathogenic role of Bacteroides.

At genus level, the abundance of Akkermansia of Phylum Verrucomicrobia was significantly increased in SS mice compared with the control. In recent years, Akkermansia become a popular genus, accounting for 83% of Phylum Verrucomicrobia, and 1–4% of the total gut microorganisms. Akkermansia is a dominant flora, and can exert many physiological functions including transformation of intestinal epithelial mucin into carbon and nitrogen sources and reduction of the thickness of intestinal mucus barrier [21]. Ganesh et al. [22] found that Akkermansia aggravated the intestinal inflammation induced by Salmonella typhimurium and destroyed the microbial balance of the intestinal mucosa of the host. It has been found that Bacteroides is related to the dysfunction of intestinal flora. When Bacteroides increases, lactobacilli decreases, thus resulting in a reduction in competitive inhibition between bacteria, growth of opportunistic pathogens, upregulation of bacterial toxins, and increased release of inflammatory factors [23]. Lactobacilli, belonging to beneficial bacteria, can ferment carbohydrates to produce lactic acid, and thus acidify the human intestinal environment, promote digestion and absorption, reduce the adhesion of harmful bacteria in the intestinal epithelium, and stimulate the body to produce a large number of immunoglobulins and strengthen the host’s immune resistance to the pathogens [24]. After combined treatment with TGP and hydroxychloroquine, Lactobacillus, Incertae, and Desulfovibrio were significantly increased, whereas Bacteroides and Alloprevotella were inhibited. Alloprevotella is a new genus of Phylum Bacteroidetes, and is a conditional pathogen, which often resides in human oral, rectal or reproductive mucosa [25]. When the body’s immune function is reduced, especially after the invasive operation or in the state of long-term immunosuppression, the chance of endogenous infection with Alloprevotella is greatly increased [26]. The beneficial effect of combined treatment with TGP and hydroxychloroquine may be because TGP can inhibit many oral pathogens, and also improve the symptoms of dry mouth, and ulcer. Further studies are required to demonstrate the mechanisms of TGP. In addition, compared with the model group, the combined drug treatment group also significantly increased the abundance of Alistipes. Alistipes is an indole-positive microorganism, which can affect the role of tryptophan. Excessive Alistipes can break the balance of the serotonin system in the gut. In addition, the combination treatment with TGP and hydroxychloroquine also increased the abundance of Incertae of Phylum Firmicutes and decreased the abundance of Pseudoflavonifractor of Phylum Firmicutes. However, the association of Incertae and Pseudoflavonifractor with SS or other diseases has not been reported in the literature. We speculate that there is a competitive relationship between Incertae and Pseudoflavonifractor in the pathogenesis of SS. Further studies are required to confirm our speculation.

## Conclusions

In summary, we performed a preliminary study to investigate the relationship between SS and gut microecology. Since the number of experimental animals is relatively small in this study, and the gut has a wide diversity of bacterial flora, we can not completely construct the intestinal ecological environment of SS patients. However, we found that the imbalance of gut bacterial flora occurred in SS by the analysis and comparison of gut bacterial flora in NOD mice vs control mice. Our findings suggest that the pathogenesis of SS is related to the imbalance of gut bacterial flora, which is consistent with our clinical study showing that SS patients exhibit intestinal microecological disorder [3]. The combination treatment with TGP and hydroxychloroquine increased the growth of many beneficial bacteria, inhibited the growth of dominant pathogenic bacteria, and increased the diversity and abundance of gut microorganisms, thus play an important therapeutic role in treating SS by improving the microecological structure of the intestinal tract and maintaining the dynamic balance of bacterial flora. Our findings provide a new experimental basis and therapeutic idea for clinical treatment of SS. Further studies are required to investigate the specific mechanisms underlying the beneficial effect of TGP and hydroxychloroquine.

## Data Availability

All data used to support the findings of this study are available from the corresponding author upon request.

## Abbreviations

TGP: Total glucoside of paeony
SS: Sjögren’s syndrome
NOD: No obesity diabetes
AQP-5: Aquaporin-5
SPF: Specific pathogen-free
OTU: Operational taxonomic unit
PCA: Principal coordinates analysis
